# Lights and Shadows of a Vegetarian Diet in Patients with Metabolic Dysfunction-Associated Steatotic Liver Disease

**DOI:** 10.3390/nu17101644

**Published:** 2025-05-12

**Authors:** Nicola Pugliese, Diletta De Deo, Matteo Soleri, Francesca Colapietro, Roberto Vettor, Alessio Aghemo

**Affiliations:** 1 Department of Biomedical Sciences, Humanitas University, 20072 Pieve Emanuele, MI, Italy; nicola.pugliese@humanitas.it (N.P.); diletta.dedeo@humanitas.it (D.D.D.); matteo.soleri@humanitas.it (M.S.); francesca.colapietro@humanitas.it (F.C.); roberto.vettor@humanitas.it (R.V.); 2 Division of Internal Medicine and Hepatology, Department of Gastroenterology, IRCCS Humanitas Research Hospital, Via A. Manzoni 56, 20089 Rozzano, MI, Italy; 3 Center for Metabolic and Nutrition Related Diseases, IRCCS Humanitas Research Hospital, Via A. Manzoni 56, 20089 Rozzano, MI, Italy

**Keywords:** MASLD, MASH, diet, vegetarian diet, vegan diet

## Abstract

The prevalence and socioeconomic impact of Metabolic dysfunction-associated steatotic liver disease (MASLD) is increasing. Despite the recent Food and Drug Administration (FDA) approval of Resmetirom as the first drug for patients with Metabolic dysfunction-associated steatohepatitis (MASH) and significant fibrosis, and several ongoing clinical trials, lifestyle changes aimed at achieving sustained weight loss remain a cornerstone in the management of these patients. In addition to regular and structured physical activity, diet is crucial. Several studies have demonstrated the benefits of the Mediterranean diet in this regard, and there is also emerging evidence on the vegetarian diet and its different patterns. This review aims to summarize the currently available evidence on the potential benefits of a vegetarian diet in patients with MASLD, as well as exploring its potential limitations.

## 1. Introduction

Metabolic dysfunction-associated steatotic liver disease (MASLD) is a significant global public health problem and is predicted to become the leading cause of liver transplantation in the coming decades [1]. It is strictly associated with obesity, type 2 diabetes (T2D), and dyslipidemia, and is characterized by hepatic fat accumulation in the presence of one or more cardiometabolic risk factors, in the absence of significant alcohol consumption or other secondary causes of liver steatosis [1,2,3]. In 2023, the term MASLD was introduced as part of an updated nomenclature with the aim of better defining this common liver disease, with a particular emphasis on metabolic dysfunction and the necessity of assessing individual metabolic syndrome components. This reclassification underscores the need for a multidisciplinary approach for MASLD management, involving hepatologists, endocrinologists, cardiologists, and nutrition specialists [4,5,6,7].

The pathophysiology of MASLD is closely linked to excess adiposity and insulin resistance, which drive hepatic fat accumulation, inflammation, and progressive liver damage. The MASLD spectrum includes simple steatosis (SLD) and metabolic dysfunction-associated steatohepatitis (MASH), the latter potentially progressing to advanced chronic liver disease (ACLD) and hepatocellular carcinoma (HCC) development [3,8,9].

In light of the global rise in metabolic syndrome, there is an expectation that the prevalence of MASLD will increase further, underscoring the need for early diagnosis, effective management strategies, and greater awareness [1,10]. The global prevalence of MASLD has risen from an estimated 25% in 2016 to over 30% today [11]. Notable regional variations have been observed, with higher prevalence rates recorded in Latin America and the Middle East compared to East Asia and Western Europe, where rates are estimated at around 25% [12].

The Food and Drug Administration’s approval of Resmetirom in 2024 is a notable advancement in the treatment of non-cirrhotic MASH. However, lifestyle modification remains the fundamental approach for managing this liver disease [7,13,14,15,16]. The primary strategy involves weight reduction through dietary modification and regular physical activity, with evidence indicating that a reduction in body weight of at least 5% is necessary to decrease liver fat, while a reduction of 7–10% is required to improve liver inflammation, and >10% to improve liver fibrosis [17].

**Figure 1:**
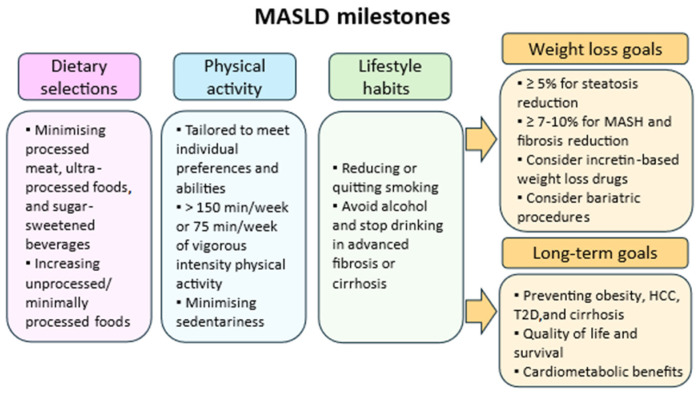
Key lifestyle milestones in MASLD management, including dietary choices, physical activity, and behavioral habits. Weight loss targets and long-term goals focus on reducing liver fat, improving fibrosis, and preventing metabolic and hepatic complications. Abbreviations: MASH (Metabolic dysfunction-associated steatohepatitis), HCC (Hepatocellular carcinoma), T2D (Type 2 diabetes).

Several dietary patterns have been explored for the treatment of MASLD, with the Mediterranean diet being the most studied and recommended [18,19]. Other plant-based dietary approaches, including the Dietary Approaches to Stop Hypertension (DASH) diet and vegetarian diet, have also demonstrated potential benefits [7,18,20,21]. The vegetarian diet, in particular, has gained attention due its potential to reduce liver fat, decrease systemic inflammation, and improve insulin sensitivity. However, despite increasing interest, evidence of its impact on MASLD remains limited. Furthermore, for individuals who do not achieve significant weight loss with lifestyle changes alone, pharmacotherapy and bariatric procedures may be considered [1,7,22,23] (Figure 1).

The present narrative review aims to summarize the currently available evidence on vegetarian dietary patterns, including their various subtypes, and evaluate their potential impact on MASLD.

## 2. Search Strategy and Inclusion Criteria

A comprehensive search of the literature was conducted to identify relevant studies on vegetarian dietary patterns in the management of MASLD. The search included major databases—PubMed, Scopus, and Web of Science—and covered publications up to March 2025. The following keywords were used: “vegetarian diet” OR “plant-based diet” OR “vegan diet” AND “MASLD” OR “NAFLD” OR “nonalcoholic fatty liver disease” OR “metabolic dysfunction-associated steatotic liver disease”. Titles and abstracts were screened for relevance, and full texts of eligible articles were reviewed. Both observational and interventional studies were considered. Inclusion criteria required studies to report clinical, biochemical, or imaging-based liver outcomes in adult human populations following vegetarian or plant-based diets (PBDs).

## 3. Vegetarian Diet: Definitions and Benefits

**Figure 2:**
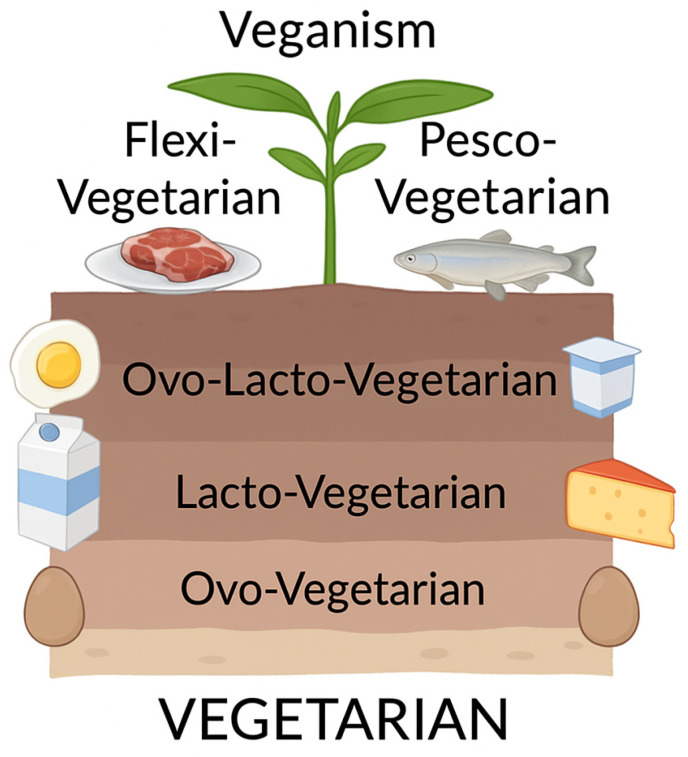
Classification of vegetarian dietary patterns.

Vegetarianism is a broad term encompassing a variety of dietary patterns that differ in their degree of restriction. It is generally defined as the exclusion of meat from the diet [24,25,26]. At the least restrictive end of the spectrum is flexitarianism, which allows occasional consumption of meat, followed by pescetarianism, where only fish and seafood are allowed. The most common form, lacto-ovo vegetarianism, excludes meat but allows eggs and dairy products. Finally, veganism, also known as strict vegetarianism, involves the elimination of all animal-derived products [24] (Figure 2).

The distribution of vegetarians around the world is not uniform. While the prevalence of vegetarianism in Western countries ranges from 4.3 to 9%, the highest proportion is found in India, where around 30% of the population follow a vegetarian diet [27,28,29,30,31].

## 4. Overall Benefits of a Vegetarian Diet

The decision to adopt a vegetarian diet is motivated by a variety of factors, the most important of which are ethical concerns and health benefits. From a health perspective, there is growing evidence of the benefits of a vegetarian diet in reducing the risk of chronic diseases, including metabolic disorders. A meta-analysis of 32 cross-sectional studies, involving over 20,000 individuals, found that vegetarian diets were associated with lower mean blood pressure compared with omnivorous diets [32]. A further meta-analysis of 15 randomized controlled trials (RCTs) involving 856 subjects supported these findings, confirming that vegetarian diets significantly reduce systolic (SBP) and diastolic blood pressure (DBP), with vegan diets having a greater effect on SBP than lacto-ovo vegetarian diets [33]. However, a separate meta-analysis of nine RCTs, involving 677 individuals, failed to find a significant association between vegan diets and blood pressure reduction, highlighting the heterogeneity observed based on dietary subtypes and study populations [34].

Several studies have shown a link between vegetarian diets and weight loss, suggesting a potential role in the management of body composition. A meta-analysis by Barnard et al. reported that prescription of a PBD was associated with a mean weight loss of 3.4 kg in an intent-to-treat analysis and 4.6 kg in a comprehensive analysis [35]. A further analysis of vegan diets and T2D found that individuals following a vegan diet had a lower prevalence and incidence of T2D, as well as improved glycemic control and glucose homeostasis [36].

Notable cardiovascular benefits were also observed. Vegan diets are rich in soluble dietary fibers (SFs), which can reduce LDL cholesterol by 5–10% without affecting HDL cholesterol or triglyceride levels [37]. This is probably due to the high viscosity of SFs, which reduces absorption of cholesterol and bile acids, and increases their fecal excretion. Moreover, PBDs also contain phytosterols (PSs), which can help to reduce cholesterol absorption by 30–40% [37]. Findings from the European Prospective Investigation into Cancer and Nutrition (EPIC)-Oxford also suggest a protective effect of a vegetarian diet against ischemic heart disease (IHD). After 18 years of observation, vegetarians had a 23% lower risk of IHD compared to meat-eaters, while vegans had an 18% lower risk [38]. When vegetarians and vegans were grouped together, the risk reduction was 22%, which remained significant at 17% after adjustment for BMI. The observed effects were probably due to lower LDL cholesterol levels and slightly lower SBP in the PBD groups. However, despite these cardiovascular benefits, vegetarians had a 17% higher risk of stroke, mainly due to a 48% increased risk of hemorrhagic stroke. Possible explanations include low LDL cholesterol levels and vitamin B12 deficiency, leading to elevated homocysteine levels [39].

With regards to cancer risk, a meta-analysis of seven studies involving 124,706 participants found that vegetarians had an 18% lower incidence of cancer compared with meat-eaters (relative risk [RR]: 0.82; 95% confidence interval [CI]: 0.67–0.97) [40]. A similar finding was reported in the EPIC-Oxford study, which examined a cohort of 65,000 subjects. This study found that the overall risk of cancer was 10% lower in vegetarians and 18% lower in vegans compared to meat-eaters [41].

## 5. Vegetarian Diet and MASLD

Vegetarian diets emphasize the consumption of fruits, vegetables, nuts, seeds, legumes, and whole grains, while minimizing animal products and processed foods [25,42]. However, several challenges need to be considered, including difficulties with adherence, the need for personalized dietary planning, and ensuring adequate nutrient intake to prevent deficiencies [43].

These diets are increasingly recognized for their potential role in the prevention and management of chronic diseases, including MASLD, where higher adherence to PBDs has been associated with a lower risk of liver steatosis, independent of genetic susceptibility [44,45].

Several studies have investigated the impact of vegetarian diets on MASLD prevalence and liver-related outcomes (Table 1).

A 2015 cross-sectional and retrospective study by Choi et al. compared 615 Buddhist monks who followed a vegetarian diet with a control group, but the results did not support a protective effect of vegetarianism against MASLD, as the prevalence was slightly higher in the vegetarian group (29.9% vs. 25.05%, *p* = 0.055) [46]. In contrast, a 2018 cross-sectional study by Chiu et al., which analyzed data from the Tzu Chi Health Study (2127 non-vegetarians and 1273 vegetarians), found that a vegetarian diet—characterized by replacing meat and fish with soy and refined carbohydrates with whole grains—was inversely associated with MASLD, BMI, and liver fibrosis severity [47]. Similarly, Mazidi et al. analyzed data from 18,345 US adults (NHANES 2005–2010) and found that adherence to a healthy PBD was associated with lower blood levels of liver enzymes (ALT, AST) and a reduced fatty liver index (FLI), while an unhealthy PBD showed the opposite trend. Participants in the highest tertile of PBD had a 21% lower odds of MASLD (OR: 0.79, 95% CI: 0.74–0.82), reinforcing the importance of PBD quality for liver health [48].

In addition to observational data, interventional studies further support the potential benefits of a vegetarian diet. A 16-week RCT, by Kahleova et al., in 244 overweight adults evaluated the effects of a low-fat vegan diet on body weight, insulin resistance, and liver fat. The intervention group showed a significant reduction in body weight (−5.9 kg, *p* < 0.001), increased postprandial metabolism (+14.1%, *p* < 0.001), and improved insulin sensitivity. There was also a 34.4% reduction in hepatocellular lipid levels (*p* = 0.002), and a 10.4% reduction in intramyocellular lipid content (*p* = 0.03) [49]. A 3-month RCT by Garousi et al. enrolled 75 overweight/obese patients with MASLD and randomized them to a lacto-ovo-vegetarian diet (LOV-D) or a standard weight-loss diet (SWL-D). Adherence to the LOV-D resulted in significant improvements in liver enzymes, anthropometric measures, glycemic markers, and lipid profiles. Moreover, the ultrasounds showed a greater reduction in liver steatosis in the LOV-D group compared to the SWL-D group [50].

The importance of diet quality was further highlighted in a longitudinal cohort study by Lv et al. that followed 159,222 participants over a median follow-up of 9.5 years. The study documented 1541 incident cases of MASLD and found that adherence to a healthy PBD was associated with a 26% lower risk of MASLD (HR 0.74, 95% CI 0.62–0.87, *p*-trend < 0.0001), independent of genetic predisposition. Conversely, an unhealthful PBD was associated with a 24% increased risk of MASLD (HR 1.24, 95% CI 1.05–1.46, *p*-trend = 0.02) [45]. Similarly, a Korean cohort study using data from the Health Examinees Study followed participants for a median of 4.2 years, during which a total of 1532 cases of MASLD were identified. The study concluded that individuals with the highest adherence to a healthy PBD had a significantly reduced risk of developing MASLD, with a risk reduction of 29% in men (HR 0.71, 95% CI 0.55–0.91, *p* = 0.0031) and 39% in women (HR 0.61, 95% CI 0.48–0.78, *p* < 0.0001), respectively [51].

These findings are further supported by a cross-sectional study by Li, X. et al. of 3900 US adults, which found that a healthy PBD was associated with 36% lower odds of MASLD (OR = 0.64, *p*-trend = 0.006). The study also found that an unhealthy PBD was associated with an increased risk of MASLD (OR = 1.37, *p*-trend = 0.009). The protective effect was strongest in non-Hispanic whites (p-interaction = 0.009) [52].

In addition, a pilot study by Chiarioni et al. investigated the effects of a personalized vegan diet in 32 MASLD patients with abnormal liver enzymes over a six-months period. The intervention resulted in a significant improvement in liver enzyme levels (*p* < 0.001 compared to baseline for AST, ALT, and GGT), despite modest weight changes. Loss of ≥5% of body weight was observed in 12 patients (46.1%), but this did not correlate with the normalization of liver function tests (*p* = 0.5), suggesting that the benefits of the vegan diet go beyond weight loss [53].

Finally, emerging evidence suggests that combining PBDs with specific dietary strategies may be useful in liver disease. A recent RCT by Shafiee et al. evaluated the combined effects of time-restricted feeding (TRF; 16/8) and LOV-D in MASLD. The intervention group showed significant reductions in body weight (−8.07 ± 4.31 kg), BMI (−2.70 ± 1.32 kg/m [2]), waist circumference (−8.00 ± 4.06 cm), ALT (−17.14 ± 14.33 U/L), GGT (−21.09 ± 24.06 U/L), FLI (−26.90 ± 15.81), insulin levels (−3.89 ± 4.69 mU/L), and TNF-α (−11.85 ± 12.52 pg/mL) (all *p* < 0.05). Improvements were also seen in the lipid profile, with lower triglycerides levels (−46.85 ± 54.55 mg/dL) and higher HDL-C (3.91 ± 5.07 mg/dL) [54].

**Table 1:** Summary of studies on vegetarian and plant-based diets in MASLD.

Author	Year	Aim	Main Findings	Study Design
Choi, S.H. et al. [46]	2015	To assess the association between vegetarianism and MASLD prevalence in a religious population.	Vegetarian diet was not protective against MASLD; slightly higher prevalence observed in vegetarians.	Cross-sectional, retrospective study of 615 Buddhist monks vs. controls.
Chiu, T.H. et al. [47]	2018	To investigate the association between vegetarian diet, liver fat, and fibrosis in healthy adults.	Vegetarian diet inversely associated with fatty liver and liver fibrosis, probably mediated by lower BMI and healthier food substitutions.	Cross-sectional study (Tzu Chi Health Study) of 3400 Taiwanese adults.
Mazidi, M. et al. [48]	2018	To evaluate associations between PBD types and MASLD/liver markers in US adults.	Inverse link between a healthy PBD and likelihood of MASLD with better liver function tests; an unhealthy PBD had the opposite effect.	Observational study (NHANES 2005–2010) of 18,345 participants.
Kahleova, H. et al. [49]	2020	To test effects of a low-fat vegan diet on liver and metabolic outcomes in overweight adults.	Vegan diet reduced body weight and liver fat, improved insulin sensitivity and postprandial metabolism.	16-week RCT with 244 participants randomized to a low-fat vegan diet or control group.
Chiarioni, G. et al. [53]	2021	To investigate the effects of a personalized vegan diet in MASLD patients with elevated liver enzymes.	Improved liver enzymes despite modest weight loss; normalization not directly related to weight change.	6-month prospective pilot study in 32 MASLD patients.
Li, X. et al. [52]	2022	To investigate the association between PBD and MASLD using transient elastography.	hPBD inversely associated with MASLD odds (OR 0.64); uPBD positively associated. Protective effect strongest in non-Hispanic whites.	Cross-sectional study (NHANES 2017–2018), 3900 US adults.
Garousi, N. et al. [50]	2023	To compare a LOV diet with a standard weight-loss diet in overweight/obese MASLD patients.	3-month LOV-D improved liver steatosis, anthropometrics, glycemia, and lipid profiles more than standard diet.	Randomized controlled trial, 75 MASLD patients.
Lv, Y. et al. [45]	2023	To investigate long-term effects of PBD quality on MASLD risk in relation to genetics.	hPBD linked to 26% lower MASLD risk; uPBD associated with increased risk. Effects independent of genetic predisposition.	Longitudinal cohort (UK Biobank), 159,222 participants, 9.5 years of follow-up.
Ulzii, B.N. et al. [51]	2024	To evaluate the association between PBD types and MASLD risk in Korean adults.	Higher adherence to hPBD reduced MASLD risk (HR 0.71 in men; 0.61 in women); uPBD associated with increased risk.	Prospective cohort study (Health Examinees Study), 1532 cases, 4.2 years.
Shafiee, M. et al. [54]	2025	To assess impact of TRF combined with LOV-D on MASLD outcomes.	TRF + LOV-D improved weight, liver enzymes, insulin, lipid profile and inflammatory markers, suggesting a viable MASLD management approach.	12-week RCT in 46 MASLD patients.

Abbreviations: MASLD, metabolic dysfunction-associated steatotic liver disease; PBD, plant-based diet index; hPBD, healthful PBD; uPBD, unhealthful PBD; LOV-D, lacto-ovo-vegetarian diet; TRF, time-restricted feeding; RCT, randomized controlled trial.

## 6. Practical Considerations for a Vegetarian Diet in MASLD Management

International guidelines recommend lifestyle modification as the primary strategy for managing MASLD [7,55,56]. The Mediterranean diet, an extensively studied dietary pattern, remains the most recommended [7,55,56]. An alternative dietary strategy for the management of MASLD is the vegetarian diet, which offers metabolic benefits due to its high fiber, antioxidant, and polyunsaturated fatty acid (PUFA) content [24]. In addition, the reduced calorie density of vegetarian diets is beneficial in the context of weight management, an important component of MASLD management [57,58].

Despite these potential benefits, the use of PBDs should be approached with caution, particularly in individuals with insulin resistance, as high intakes of carbohydrates from certain plant sources may lead to the accumulation of liver fat. In addition, the use of dietary planning and supplementation to address nutrient deficiencies, particularly vitamin B12, *n*-3 PUFAs, and iron, is recommended. It is noteworthy that studies have found B12 deficiency in up to 50% of vegans, highlighting the importance of fortified meals and regular monitoring to prevent adverse effects [59]. An altered *n*-6/*n*-3 ratio has been observed in patients with MASLD, suggesting that optimizing *n*-3 PUFA intake through diet or supplementation may further improve liver health [7].

From a practical standpoint, the provision of tailored dietary advice and organized patient education is essential for ensuring long-term adherence to a vegetarian diet. To ensure a balanced macronutrient profile and to avoid excessive reliance on refined carbohydrates, it is recommended that MASLD patients be supported by healthcare professionals, such as dietitians and nutritionists, in selecting nutrient-dense plant-based diets.

## 7. Conclusions

Pending the availability of Resmetirom in Europe for patients with non-cirrhotic MASH and the results of ongoing phase 3 trials, dietary interventions remain the first line of therapy for the management of patients with MASLD, particularly with the goal of achieving significant weight loss, as this can define an improvement in liver histology [7,13]. Although no firm conclusions can be drawn on the relative merits of vegetarian versus Mediterranean diets in terms of outcomes in patients with MASLD, it is clear that these dietary patterns have distinct advantages in the management of obesity, metabolic dysfunction, and cardiovascular risk factors. The development of personalized dietary recommendations that integrate genetic predisposition and biomarker-based approaches is a key area for future research with the aim of optimizing PBD interventions for patients with MASLD. Artificial intelligence is also emerging as a potentially useful tool for patients with MASLD. In particular, AI-based chatbots could be used as a tool that patients can turn to at any time to ask questions about their dietary regimen, potentially improving adherence and the likelihood of achieving treatment goals [60,61]. The integration of traditional and innovative resources and the holistic approach to the patient with MASLD are certainly cornerstones in the management of this increasingly prevalent disease.

## Data Availability

No new data were created or analyzed in this study.
